# A Case of Refractory Thyroid Storm Despite Correction of Peripheral Thyroid Hormone Levels

**DOI:** 10.1210/jcemcr/luae179

**Published:** 2024-10-16

**Authors:** Madeline Evans, Grace Prince, Priyanka Majety

**Affiliations:** Virginia Commonwealth University Department of Internal Medicine, Richmond, VA 23298, USA; Division of Endocrinology, Diabetes and Metabolism, Virginia Commonwealth University Department of Internal Medicine, Richmond, VA 23298, USA; Division of Endocrinology, Diabetes and Metabolism, Virginia Commonwealth University Department of Internal Medicine, Richmond, VA 23298, USA

**Keywords:** thyroid storm, severe thyrotoxicosis, refractory thyroid storm

## Abstract

Thyroid storm is a life-threatening complication of hyperthyroidism that necessitates early diagnosis for aggressive, effective treatment. We present a patient with a newly diagnosed multinodular goiter who presented to the emergency department with leg swelling, dyspnea, tremors, and atrial fibrillation with elevation in thyroid hormone levels consistent with thyrotoxicosis. Despite improvement in peripheral hormone levels on maximized medical treatment with beta-blockers, methimazole, glucocorticoids, cholestyramine, and potassium iodide, she continued to clinically decline with new encephalopathy, heart failure, and liver and kidney dysfunction while receiving treatment. Work-up for alternative causes of her clinical decompensation was unrevealing. Plasmapheresis was initiated, with further reduction in thyroid hormone levels without clinical improvement. Cases in the literature do report incidences of severe thyrotoxicosis refractory to traditional treatment measures; however, generally, these cases involve a failure to reduce thyroid hormone levels with medical treatment and subsequent consideration of plasmapheresis. Our case suggests that clinical improvement in thyroid storm does lag behind biochemical improvement in select patients, and delayed clinical improvement or even severity of symptoms may warrant earlier consideration of plasmapheresis in such patients.

## Introduction

Thyroid storm is a rare and serious complication of thyrotoxicosis associated with 10% to 30% mortality [1, 2]. Thyroid storm typically presents with traditional symptoms of hyperthyroidism as well as signs of end-organ damage including cardiovascular compromise (eg, tachyarrhythmias, heart failure), hepatic injury, gastrointestinal distress, and neurologic changes ranging from coma to psychosis. In most cases, thyroid storm occurs either in patients with poorly controlled hyperthyroidism or in patients with hyperthyroidism in the setting of a precipitating event such as infection, surgery, myocardial injury, parturition, or iodine administration. While peripheral thyroid hormone elevation is essential in the diagnosis of thyrotoxicosis, the degree of biochemical elevation does not correlate with the severity of disease and is not used to diagnose thyroid storm [3]. However, the improvement of peripheral thyroid hormone levels does typically correlate with clinical improvement [4]. Here we present a case of thyroid storm characterized by progressive clinical decline despite aggressive medical treatment and biochemical improvement in thyroid hormone levels.

## Case Presentation

A 56-year-old female with obesity, hypertension, and newly diagnosed hyperthyroidism presented to the emergency room with leg swelling, dyspnea, tremor, weight loss, and palpitations for 4 months. History was significant for a diagnosis of multinodular goiter and hyperthyroidism 3 months prior to presentation, for which she had not yet started treatment, and a recent sinus infection treated with amoxicillin.

## Diagnostic Assessment

At presentation, she was in atrial fibrillation with rapid ventricular rate (RVR) with a heart rate up to 140 beats per minute though other vitals were within normal range. On exam, she was alert and oriented with palpable thyromegaly and diffuse nodularity without thyroid bruit, bilateral lower extremity edema, and diaphoresis. She was able to provide appropriate history, and family members confirmed that no changes in mental status had been noted prior to presentation. Initial laboratory findings were significant for thyroid function tests consistent with uncontrolled hyperthyroidism [TSH <0.01 μIU/L (normal reference range 0.36-3.74 μIU/L), free T4 6.4 ng/mL (82 pmol/L) (normal reference range 0.7-1.5 ng/mL; 9-19.3 pmol/L), total T4 >24 μg/dL (>309 nmol/L) (normal reference range 4.9-11.7 μg/dL; 63-151 nmol/L), free T3 17.1 pg/mL (0.26 pmol/L) (normal reference range 2.2-4.0pg/mL; 0.034-0.062 pmol/L)]. Electrocardiogram confirmed atrial fibrillation with RVR with mild right heart strain on bedside ultrasound [N-terminal pro b-type natriuretic peptide 918 pg/mL (109 pmol/L) (normal reference range <125 pg/mL; <15 pmol/L)]. Computed tomography scan without contrast and thyroid ultrasound revealed multinodular symmetric goiter without airway compromise.

## Treatment

**Figure 1.**
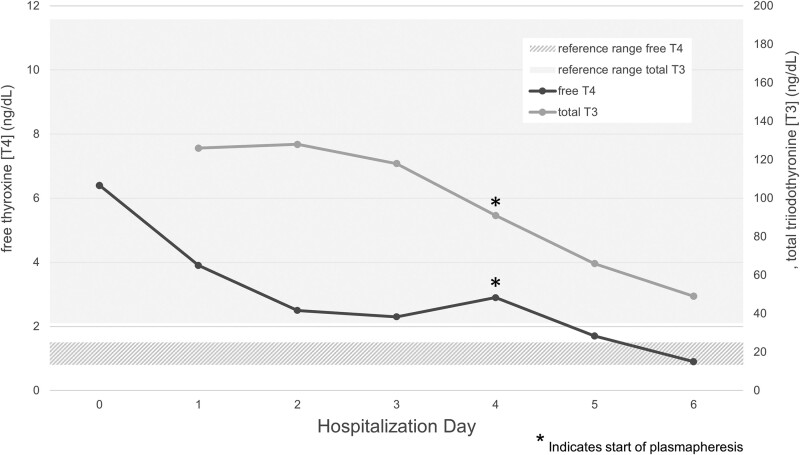
Thyroid hormone levels during hospitalization.

She was started on hydrocortisone 100 mg every 8 hours, propylthiouracil (PTU) 200 mg every 4 hours, and an esmolol infusion and admitted to the intensive care unit. As seen in Fig. 1, within 24 hours of initiating treatment, her thyroid function tests were improving as expected [free T4 2.5 ng/mL (32 pmol/L), total T3 128 ng/dL (1.97 nmol/L), (normal reference range 35-193 ng/dL; 0.5-2.96 nmol/L)]. However, despite improvement in peripheral hormone levels, she developed new-onset heart failure with liver and kidney injury. PTU was switched to methimazole 20 mg every 6 hours to reduce the risk of worsening hepatotoxicity, esmolol infusion was transitioned to propranolol 80 mg every 4 hours to reduce additional volume, and potassium iodide 250 mg every 6 hours and cholestyramine 4 g every 6 hours were added as adjunctive therapy. She remained tachycardic despite maximum doses of beta-blockade with worsening hypervolemia and laboratory testing concerning for progressive multiorgan failure. Additionally, she showed new neuropsychiatric symptoms of hypoactive delirium and agitation. Initially, she could follow commands and respond to verbal stimuli, but within 24 hours of encephalopathy onset, she was endotracheally intubated for airway protection and a nasogastric tube was placed as she was only responsive to pain.

Due to her progressive clinical decline, plasmapheresis was initiated, though hormone levels had improved [free T4 2.9 ng/dL (37.3 pmol/L), total T3 91 ng/dL (1.4 nmol/L)]. Two sessions of plasmapheresis did further improve her peripheral hormone levels [free T4 0.9 ng/dL (11.6 pmol/L), total T3 48 ng/dL (0.74 nmol/L)], but she remained in atrial fibrillation with RVR despite maximized treatment and went on to require vasopressors for cardiovascular compromise and continuous renal replacement therapy for renal failure. She remained unresponsive without sedation and failed several spontaneous breathing trials.

Alternate causes of multiorgan failure and encephalopathy were considered, and a thorough workup, including electroencephalogram, brain imaging, infectious workup, and metabolic analysis, was unrevealing. Ultimately, symptom burden was attributed to her underlying thyrotoxicosis despite appropriate treatment and lab improvement.

## Outcome and Follow-up

Several days into hospitalization, while undergoing intensive medical therapy, the patient's autoimmune laboratory testing revealed elevated thyrotropin receptor antibody (3.02 IU/L; normal reference range 0.00-1.75 IU/L) and thyroid-stimulating immunoglobulin (2.77 IU/L; normal reference range 0.00-0.55 IU/L) consistent with a diagnosis of Graves disease. Unfortunately, despite plasmapheresis and maximal medical management, she suffered a cardiac arrest and was not able to be resuscitated.

## Discussion

Thyroid storm is a rare but serious complication of thyrotoxicosis. Given the high rate of mortality, timely and effective diagnosis and treatment aimed at reducing active thyroid hormone is imperative.

While the degree of peripheral hormone elevation does not necessarily correlate with severity of disease, improvement of peripheral thyroid hormones does typically correlate with clinical improvement. Pharmacologically this is accomplished by blocking synthesis (PTU, methimazole), secretion (potassium iodide), circulation (cholestyramine), and conversion (beta-blockade, steroids) of thyroid hormone. In most cases, this treatment is effective in reducing symptom burden and hormone levels with very few reported cases of refractory thyrotoxicosis [5]. This patient presents a unique case of continued clinical decline despite what otherwise appeared to be effective medical therapy based on biochemical parameters.

While there are rare cases in the literature of thyrotoxicosis refractory to these traditional treatment measures, most cases also report a failure of peripheral hormone levels to respond to the aggressive treatment [6-8]. In these cases, it is certainly appropriate to consider plasmapheresis to rapidly remove peripheral hormones as a bridge to thyroidectomy [9]. By exchanging the patient's plasma along with circulating protein-bound thyroid hormones and autoantibodies for albumin or fresh frozen plasma, plasmapheresis rapidly reduces peripheral thyroid hormone levels. There is very limited data to guide further management of thyroid storm when symptoms worsen despite reduction of peripheral hormone levels with traditional treatment measures, as was seen in our patient. In these cases, it is necessary to investigate alternate etiologies for the clinical decline. However, if workup is unremarkable, plasmapheresis could still be considered to further, and more quickly, reduce peripheral hormone levels.

An additional point of interest in this case is the neuropsychiatric decline that occurred after improvement of hormone levels. Central nervous system compromise in thyrotoxicosis is well established and includes a range of presenting symptoms from psychosis to coma. There are several proposed mechanisms of encephalopathy in thyroid storm including several interactions between thyroid hormones and neurotransmitter circuits [10]. Hyperthyroidism has been linked to increased β-receptor-mediated adrenergic activity and sodium current in the brain, both of which increase neuronal excitability [11, 12]. Additionally, TSH receptors are prevalent in certain areas of the brain, particularly the hippocampus and cortex [13]. In Graves disease, the antibodies activate the TSH receptors, increasing local production of T3 and thereby contributing to psychiatric symptoms [14, 15].

While the mechanisms underlying neurologic decline in thyroid storm are still debated, the development of these symptoms in our patient with Graves disease is not unexpected. Most concerning in this case, however, was the onset of neurologic symptoms after the reduction in peripheral hormone levels and further decline once labs normalized. While data does support the use of plasmapheresis in cases of thyroid storm in which peripheral hormone levels do not improve with traditional medications, there are no reported cases of plasmapheresis use in patients with appropriate biochemical improvement with medical therapy but further clinical decompensation thereafter. This case suggests that clinical improvement may lag behind biochemical improvement in select patients with thyroid storm, and it may highlight the need for earlier consideration of plasmapheresis in such patients. Indications and guidelines for the use of early plasmapheresis should be considered and defined to decrease mortality associated with thyroid storm.

## Learning Points

- Thyroid storm is a rare yet serious complication of thyrotoxicosis, typically marked by traditional symptoms of hyperthyroidism as well as signs of end-organ involvement and hemodynamic compromise (eg, fever, tachyarrhythmia, central nervous system disturbance, gastrointestinal symptoms).
- Peripheral thyroid hormone elevation is a hallmark of thyrotoxicosis, but the degree of biochemical elevation does not correlate with disease severity and it is not used to diagnose thyroid storm.
- Improvement in peripheral thyroid hormones typically correlates with clinical improvement. For the rare cases in which it does not, further workup for alternate etiologies of a patient's declining condition should be undertaken.
- Refractory cases of thyroid storm may respond to plasmapheresis, which should be considered even in patients with improving peripheral hormones but worsening clinical features of thyroid storm.

## Contributors

All authors made individual contributions to authorship. All authors contributed to initial patient management. G.P. and P.M. were key subspecialty consultants in diagnosis and treatment. M.E. was part of the patient's primary inpatient team while admitted to intensive care. All authors reviewed and approved the final draft.

## Data Availability

Data sharing is not applicable to this article as no datasets were generated or analyzed during the current study.
